# Impaired Spatial Inhibition Processes for Interhemispheric Anti-saccades following Dorsal Posterior Parietal Lesions

**DOI:** 10.1093/texcom/tgab054

**Published:** 2021-09-13

**Authors:** Julie Ouerfelli-Ethier, Romeo Salemme, Romain Fournet, Christian Urquizar, Laure Pisella, Aarlenne Z Khan

**Affiliations:** School of Optometry, University of Montreal, Montreal H3T 1P1, Canada; Lyon Neuroscience Research Center, Trajectoires Team, INSERM 1028, CNRS UMR 5292, University of Lyon I Claude-Bernard, Lyon 69500, France; Lyon Neuroscience Research Center, Trajectoires Team, INSERM 1028, CNRS UMR 5292, University of Lyon I Claude-Bernard, Lyon 69500, France; School of Optometry, University of Montreal, Montreal H3T 1P1, Canada; Lyon Neuroscience Research Center, Trajectoires Team, INSERM 1028, CNRS UMR 5292, University of Lyon I Claude-Bernard, Lyon 69500, France; Lyon Neuroscience Research Center, Trajectoires Team, INSERM 1028, CNRS UMR 5292, University of Lyon I Claude-Bernard, Lyon 69500, France; School of Optometry, University of Montreal, Montreal H3T 1P1, Canada

**Keywords:** eye movements, optic ataxia, priority maps, remapping

## Abstract

Anti-saccades are eye movements that require inhibition to stop the automatic saccade to the visual target and to perform instead a saccade in the opposite direction. The inhibitory processes underlying anti-saccades have been primarily associated with frontal cortex areas for their role in executive control. Impaired performance in anti-saccades has also been associated with the parietal cortex, but its role in inhibitory processes remains unclear. Here, we tested the assumption that the dorsal parietal cortex contributes to spatial inhibition processes of contralateral visual target. We measured anti-saccade performance in 2 unilateral optic ataxia patients and 15 age-matched controls. Participants performed 90 degree (across and within visual fields) and 180 degree inversion anti-saccades, as well as pro-saccades. The main result was that our patients took longer to inhibit visually guided saccades when the visual target was presented in the ataxic hemifield and the task required a saccade across hemifields. This was observed through anti-saccades latencies and error rates. These deficits show the crucial role of the dorsal posterior parietal cortex in spatial inhibition of contralateral visual target representations to plan an accurate anti-saccade toward the ipsilesional side.

## Introduction

Anti-saccades are eye movements directed to a location opposite to a presented stimulus (Hallett 1978; Mokler and Fischer 1999; Munoz and Everling 2004). The successful execution of an anti-saccade mainly relies on 2 subprocesses: 1) the inhibition of the automatic saccade to the visual target and 2) the generation of an anti-saccade away from the target (Munoz and Everling 2004; Hutton 2008).

Previous studies have implicated the involvement of multiple types of inhibition in stopping the automatic saccade during anti-saccades. Preparatory set or proactive inhibition refers to top-down inhibition that is present before the stimulus appears and entails a global inhibition from making an eye movement (Funahashi et al. 1993; Everling and Munoz 2000; Barash and Zhang 2006; Sharpe et al. 2011; Coe and Munoz 2017; Jahanshahi and Rothwell 2017; Fernandez-Ruiz et al. 2018). This top-down inhibition appears to be nonspatial, suppressing automatic saccades to the visual target regardless of its location (Guitton et al. 1985; Barash and Zhang 2006) and nonspecific to anti-saccades; pro-saccade latencies are also increased during interleaved pro/anti-saccade tasks (Cherkasova et al. 2002; Ansari et al. 2008; Ethridge et al. 2009; Zeligman and Zivotofsky 2017; Ayala and Niechwiej-Szwedo 2021). A second type of inhibition involved in anti-saccades is response inhibition, which is defined as the requirement to cancel a previously generated motor plan (Cutsuridis, Smyrnis, et al. 2007b). Response inhibition is considered to be reactive in that it is a response to an external stimulus (Cutsuridis, Smyrnis, et al. 2007b; Jahanshahi and Rothwell 2017).

Anti-saccades also entail making a voluntary saccade to a location in which there is no stimulus. This has been presumed to be achieved via a vector inversion process, whereby the visual vector is inverted to create a new motor plan (Zhang and Barash 2000; Munoz and Everling 2004; Zhang and Barash 2004; Collins et al. 2008; Blangero et al. 2011; Lévy-Bencheton et al. 2013) as well as competitive activation and inhibition between the 2 possible locations (i.e., anti-saccade goal and visual target), which recruit spatial inhibition processes. Spatial inhibition serves to bias the competition between anti-saccade goal and visual target by dampening the neuronal activity associated with the location of the latter (Zhang and Barash 2000; Zhang and Barash 2004). This competition has been considered to take place in a type of winner-take-all attentional priority map to reach a decision threshold of where the eyes should go (Findlay and Walker 1999; Mokler and Fischer 1999; Smyrnis et al. 2002; Massen 2004; Munoz and Everling 2004; Kristjansson 2007; Cutsuridis, Kahramanoglou, et al. 2007a; Cutsuridis, Smyrnis, et al. 2007b; Noorani and Carpenter 2013; Zelinsky and Bisley 2015) and is thus highly linked to spatial attention. Specifically, there is a spatially specific inhibition/suppression at one location, for example, the inhibition of the visual target (Zhang and Barash 2000; Zhang and Barash 2004; McSorley et al. 2006; Dhawan et al. 2013), caused by excitation (attention) at another location, for example, saccade goal (Everling et al. 1998), potentially implemented through lateral connectivity such as in the superior colliculus (Munoz and Fecteau 2002; Khan et al. 2016). Behaviorally, a lack of spatial inhibition would result in greater error rates (ERs) for anti-saccades in which the visual target location was not sufficiently inhibited, for example, due to damage to connectivity, rather than overall increased ERs regardless of location, more associated with response inhibition.

Overall, the different inhibitory processes involved in anti-saccades have been attributed to the frontal cortex (Guitton et al. 1985; DeSouza et al. 2003; Munoz and Everling 2004; Hutton 2008); anti-saccade deficits have been demonstrated in numerous clinical populations with frontal dysfunction such as Parkinson’s disease (Hood et al. 2007; Cameron et al. 2010; Antoniades et al. 2015), schizophrenia (Klein et al. 2000; McDowell et al. 2002), and attention deficit disorder (Klein et al. 2003). Tasks that measure response inhibition, such as the countermanding task and the go/no-go task, have also been shown to involve frontal areas (Casey et al. 1997; Menon et al. 2001; Aron et al. 2004; Swick et al. 2008; Middlebrooks et al. 2017). Similarly, imaging and neurophysiological studies have highlighted the role of eye movement areas such as the dorsolateral prefrontal cortex and frontal eye fields in anti-saccade inhibition, response inhibition, and competition (Funahashi et al. 1993; Everling and Munoz 2000; Klein et al. 2000; Hutton and Ettinger 2006; Heath et al. 2016; Fernandez-Ruiz et al. 2018).

The distinct role of the parietal cortex in inhibition from that of the frontal cortex remains unclear. Patients with hemispatial neglect whose main symptom is a lack of attentional awareness of the contralesional side (Vallar 1998; Kerkhoff 2001; Rode et al. 2017), usually the left side after right inferior parietal lobule (IPL) damage, show evidence for response inhibition deficits; they have “bilateral” anti-saccade deficits consecutive to unilateral brain damage with overall higher anti-saccade ERs and latencies (Butler et al. 2009; Sharpe et al. 2011). This behavior is similar to patients with frontal dysfunction who show response inhibition deficits (Guitton et al. 1985; Klein et al. 2000; McDowell et al. 2002; Klein et al. 2003; Hood et al. 2007; Cameron et al. 2010; Antoniades et al. 2015).

Dorsal parietal cortex damage, which is well known to be associated with deficits in visually guided movements (i.e., optic ataxia; review in Pisella et al. 2021), is also associated with spatial attention deficits (Striemer et al. 2007; Khan et al. 2009; Striemer et al. 2009; Blangero et al. 2011; Gillebert et al. 2011; Khan et al. 2016). Typically, both visually guided movement deficits and spatial attentional deficits tend to occur in the contralesional hemifield for left as well as right brain-damaged patients; and bilateral deficits are observed in bilateral damaged patients (Jax et al. 2009; Khan et al. 2009; Gillebert et al. 2011; Pisella et al. 2011; Khan et al. 2016; Mikula et al. 2021). The dorsal parietal cortex therefore plays a central role in spatial attention. However, it remains unknown what role the dorsal parietal cortex plays in spatial inhibition. It has been suggested to be involved in representing the priority map underlying competition as well as spatial inhibition (Bisley and Goldberg 2010; Mirpour et al. 2010; Ptak 2012).

Thus, testing unilateral optic ataxia (deficits within the hemifield space opposite to the damaged hemisphere) offers considerable insight into the mechanisms subtending anti-saccade production as it allows for the characterization of specific contralesional spatial inhibition deficits. This can be achieved because such deficits involve damage to brain areas implicated in priority maps (Pisella et al. 2007; Striemer et al. 2007; Pisella et al. 2009; Striemer et al. 2009). Unlike with hemispatial neglect (following damage to right IPL), response inhibition deficits have not been observed during reaching movements in optic ataxia (following dorsal parietal cortex damage). For example, a bilateral optic ataxia patient was able to interrupt online movements as fast as controls in contrast to a patient with dorsolateral frontal cortex (Pisella et al. 2000). Further, optic ataxia patients tend to make more errors in anti-pointing tasks when the target is in their contralesional field regardless of movement direction (Blangero et al. 2011), highlighting possible deficits in spatial inhibition and vector inversion processes. Taken together, in optic ataxia, both spatial inhibition and inversion processes appear to be impaired in the contralesional side and linked to their visual attention deficits. It has also been hypothesized that vector inversion processes for anti-saccades are calculated in the intraparietal sulcus (Zhang and Barash 2000; Zhang and Barash 2004) where mental rotation activity has been revealed, crucially involving the right hemisphere (Harris and Miniussi 2003; Schendan and Stern 2007).

Here, we tested the hypothesis that the dorsal parietal cortex may play a specific role in spatial inhibition related to vector inversion and competition. We therefore tested 2 patients with unilateral optic ataxia in various versions of the anti-saccade task, comprising pro-saccades, across anti-saccades (90° away across hemifields), within anti-saccade (90° away within the same hemifield), and classic anti-saccades (180° away). These versions allowed us to test for spatially specific inhibitory, vector inversion, and competition processes by comparing anti-saccade performance across the 2 hemispheres (damaged and intact).

## Materials and Methods

### Participants

We recruited 2 patients with parietal lesions presenting optic ataxia via the neurological and rehabilitation hospitals, Lyon, France. Fifteen controls were recruited from the community in both Lyon and Montreal.

**Figure 1:**
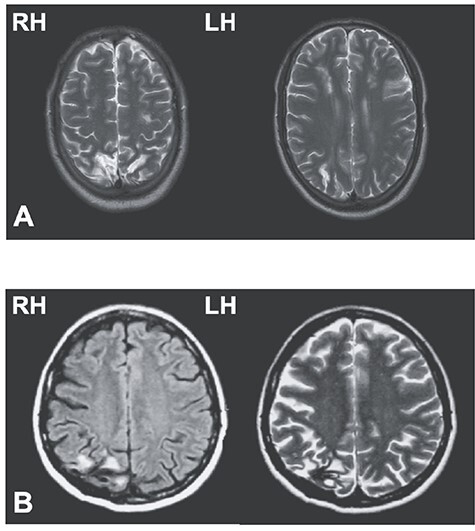
Lesions of patients C.F. and M.L. In (*A*), T1 anatomical scan of patient C.F.’s bilateral posterior parietal lesion, with larger damage in the right hemisphere. In (*B*), T1 (left panel)- and T2 (right panel)-weighted horizontal magnetic resonance imaging shows M.L.’s unilateral lesion in the posterior parietal cortex of the right hemisphere (black area in T1 and white area in T2). LH, left hemisphere; RH, right hemisphere.

Patient C.F. is a right-handed 33-year-old male who suffered from a watershed posterior infarct, 10 years before testing. The infarct resulted in distributed and asymmetrical bilateral lesions of the occipito-parietal region (Brodmann’s areas 18, 19, 7, 5, and 2) with a minute extension to the semiovale centers. At the time of testing, he exhibited optic ataxia in his left visual field, thought to be the consequence of larger damage in the right hemisphere from both BA 7 lesions and a parieto-frontal disconnection from intra-hemispheric fibers lesions—(Fig. 1*A*, see also Khan et al. 2005). He did not exhibit any purely motor, somatosensory, or visual deficits or any sign of neglect shown through a set of standard clinical tests involving visual field topography, sensory stimulation tests, evaluation of reflexes and muscle tone, and joint movements.

Patient M.L. is a left-handed 60-year-old female, who suffered from a hemorrhagic stroke in the right hemisphere, 18 years before testing. The lesion damaged the parieto-occipital junction as well as the caudal parts of both the intraparietal sulcus and of the superior parietal lobule (Fig. 1*B*). Following this focal lesion, M.L. exhibited optic ataxia symptoms isolated to the left visual field using both hands (Blangero et al. 2011).

For each of our patients, we recruited control participants age-matched within 5 years (C.F.’s controls: *N* = 6, age range = 26–35 years, *M* = 30.7 years, SD = 2.7 years, 3 women; M.L.’s controls: *N* = 9, age range = 61–75 years, *M* = 65.4 years, SD = 4.9, 6 women). Control participants with neurological disorders or attentional deficits were excluded. All participants had normal or corrected to normal vision and gave informed written consent to participate in the experiment. Procedures were conformed according to the French law (4 March 2002) on human subjects’ rights and received ethics approval in Lyon and from CERC at the University of Montreal.

### Apparatus

Testing occurred at the University of Montreal (Montreal, Canada) and at the Centre of Neuroscience Research of Lyon (CNRL; Lyon, France) with similar apparatus for eye-movement recording. Participants sat in a dark room 57 cm away from a high-speed computer screen (at CNRL: 15.7*11.8 inches, Visual Stimulus Generator ViSaGe, Cambridge Research System, Rochester, UK; at the University of Montreal, 20.5*11.5 inches, VIEWpixx 3D, VPixx Technologies, Montreal, Canada). Head movements were restricted with chin and forehead rests during the task. An eye-tracker, set in a binocular tower-mount, recorded eye movements (at CNRL: ViSaGe, Cambridge Research System, Rochester, UK, frequency: 250 Hz; at the University of Montreal: EyeLink 1000 Plus, SR Research, Kanata, Canada, frequency: 1000 Hz).

### Procedure

**Figure 2:**
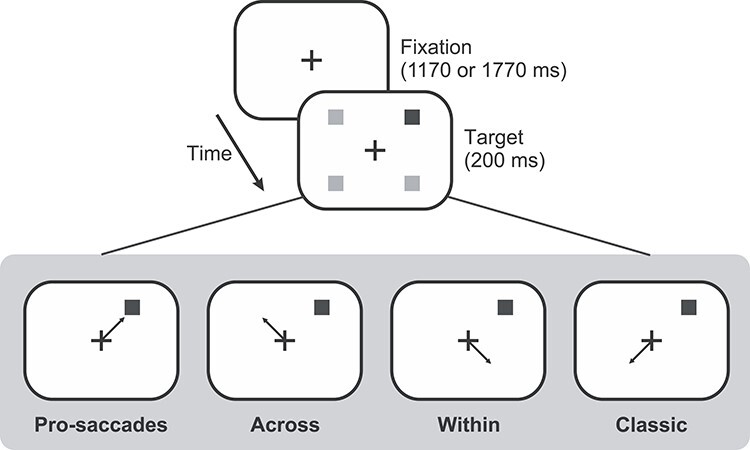
Experimental sequence and timings of the 4 conditions of the saccade task. The fixation cross remained on the screen for 1170 or 1770 ms. The target (black square) appeared for 200 ms while fixation cross remained on for an additional 1000 ms after target appearance. For all panels, correct saccades according to target are illustrated by an arrow while gray squares represent possible target locations. After the fixation cross disappeared, there was an inter-trial interval (ITI) of 100 ms.

As shown in Figure 2, participants performed saccades in 4 paradigms: 1) pro-saccades, 2) mirror saccades (90° rotation) “across” (horizontal) visual fields, 3) mirror saccades (90° rotation) “within” (horizontal) visual fields, and 4) classic anti-saccades (180° rotation). Tasks were designed and implemented using Matlab (The MathWorks, Inc., Natick, MA) with the Psychophysics toolbox (Brainard 1997).

During pro-saccades, participants made a saccade towards the target as quickly as possible when it appeared. In the across condition, participants were asked to inhibit a saccade toward the target and to make, instead, a saccade 90° away from it across the other hemifield. For the within condition, participants made a saccade 90° away from target vertically or within the same hemifield. Finally, during the classic condition, participants gazed 180° away from visual target.

All paradigms consisted of the same stimuli presentation as follows: Each trial began with the presentation of white fixation cross (dimensions: 1° by 1°) centered on the screen against a black background. The fixation cross was aligned horizontally with the midpoint of the eyes and vertically at eye level. The fixation cross was present throughout the trial. After a variable duration (1170 or 1770 ms), a target was presented at 1 of 4 oblique locations (Fig. 2) at a distance of 7.78° from the fixation cross (5.5° horizontally and vertically). The target was a gray square with a diameter of 1° and was presented for 200 ms. An auditory beep was sounded at the same time as the target as a cue for the participants to make a saccade. The fixation cross remained illuminated for an additional 1000 ms and then was followed by a blank screen for 100 ms signaling the next trial. Target presentation was pseudorandom across 4 possible target locations.

The 4 paradigms were performed in blocked order. As shown in Figure 2, for the pro-saccade paradigm, participants were instructed to make a saccade to the target location as soon as they saw the target (black arrow indicates the correct direction of the saccade). For the across paradigm, participants made a saccade rotated 90° from the target location in the same vertical but to the opposite horizontal direction. For the within paradigm, they made a saccade rotated 90° in the same horizontal but the opposite vertical direction. For the classic paradigm, participants were asked to make a saccade to the location 180° away from the target as soon and as accurately as possible. Patients and controls were given the same instructions, and they all indicated that they understood the task.

Participants performed all 4 paradigms in blocks within the same session or across 2 sessions and the paradigm order was counterbalanced across participants. Patient C.F. completed 88 trials for the pro-saccade paradigm and 160 trials for each of the 3 other paradigms. Patient M.L. completed 88 trials each for all paradigms except for the classic paradigm where she completed 288 trials. Control participants each performed between 80 and 244 trials per paradigm. One control participant did not perform the classic anti-saccade paradigm. The number of trials varied due to time and patient errors/constraints.

### Preliminary Analyses

We recorded a total of 6634 trials. To account for the different sampling rates from the 2 cameras, we extracted data separately using scripts tailored to each sampling rate to ensure timings were preserved. Thereafter, data were analyzed in the same manner. We did not encounter any limitations for our analyses as we ensured very similar setups in both testing centers. Saccade timing and position were automatically calculated offline using a saccade detection algorithm with a velocity criterion of 15°/s and verified visually. Manual inspection involved removing trials in which saccades were made before the target appeared, there was a blink during the saccade, or the tracker lost the eye position. Following this, one of M.L.’s controls had an insufficient total number of trials remaining (49 trials), so they were removed from further analyses (*n* = 8). For the remaining participants, we removed all trials with blinks, which were automatically recorded as saccades with endpoints greater or less than 1000° in *x* and *y* positions (17 trials, 0.26% of total number trials).

After removing these outliers, we normalized start positions per participant according to their mean start position for their eyes in *X* and *Y*. We filtered out start positions beyond 3 SDs from each participant’s mean (34 trials, 0.51% of total number of trials). Next, we removed all trials with saccade reaction times (SRTs) below 100 ms to exclude anticipatory and express saccades (Fischer and Ramsperger 1984; Fischer and Ramsperger 1986; Mayfrank et al. 1986; Weber et al. 1992; Fischer and Weber 1997) (198 trials, 2.98% of total number of trials). For the remaining 6385 trials, we filtered out per participant all trials with SRTs outside of 3 SDs of their mean (65 trials, 0.98% of total number of trials). All trials with a first saccade amplitude of smaller than 2° were also removed (551 trials, 8.31% of total number of trials). There remained 5789 trials (87.3% of total number of trials).

We calculated ERs as the percentage of erroneous anti-saccades for all anti-saccade trials; an erroneous anti-saccade was considered to be one in which the first saccade after the target presentation that was directed toward the visual target. Specifically, the endpoint landed within the quadrant of the visual target (excluding 10° of the cardinal directions). In contrast, correct anti-saccades were defined as saccades landing in the saccade goal quadrant (excluding 10° of the cardinal directions). For example, a classic anti-saccade would be considered erroneous for a visual target presented at 45° if it landed between 10 and 80° (polar coordinate system). For this visual target, the saccade goal is located at 225°, so any saccade with an endpoint between 190 and 260° would be considered correct. Saccades that were not directed to the visual target or saccade goal were not considered in the analyses. SRTs were obtained by subtracting target onset from saccade onset for correct saccades. We compared ERs and SRTs for each condition.

We furthered these analyses by investigating anti-ERs as a function of SRTs. All anti-saccade trials were collapsed across conditions; patients did not have enough trials to do this analysis per condition. SRTs were then binned in 50 ms increments from 75 to 1500 ms. We fitted psychometric curves for each participant’s ER as a function of SRTs using the psignifit 3.0 toolbox with the Bayesian Inference fitting procedure (Fründ et al. 2011) and Matlab (Mathworks, Natick, MA) to Gaussian sigmoid functions. No priors were imposed for the mean or slope of the function. The priors of the upper and lower thresholds were set to 0.1, to account for lapses. From the psychometric curves, we obtained 20% thresholds values for each participant, that is, the SRT at which each participant’s ERs had decreased to 20%. The thresholds were obtained separately per hemifield for both patients along with their controls.

We also examined spatial errors in correct saccade endpoints looking at absolute errors and saccade endpoints. Absolute errors were calculated by subtracting each saccade endpoint from target position (in pro-saccades), or saccade goal (in anti-saccades), for all participants in absolute values of visual degrees for correct saccades. Saccade endpoints were averaged for each possible target position, per condition and per participant in both *X* and *Y* dimensions.

To verify that participants understood task instructions, we calculated the percentage of erroneous anti-saccade corrected with a second saccade.

We used modified *t*-tests to compare anti-saccade performance between each patient and their control group (Crawford and Howell 1998; Crawford and Garthwaite 2002; Crawford et al. 2010). As C.F. and M.L. presented with unilateral optic ataxia, we separately tested how left and right visual targets affected anti-saccade performance for the above parameters with the modified *t*-test method described previously. We additionally compared the difference between hemifields for each patient and their control group with a test for difference between 2 *t*-variates (Crawford and Garthwaite 2005; Crawford et al. 2010) to highlight differences between affected and unaffected hemifields.

## Results

We examined ERs and SRTs to compare task performance across conditions between patients and their controls. We followed this analysis by comparing ERs as a function of SRTs to investigate whether patients may have adopted a strategy where they slowed their response to ensure low ERs. We also investigated the accuracy of correct saccades (spatial errors at saccade endpoints) for all participants.

### Raw Trajectories

**Figure 3:**
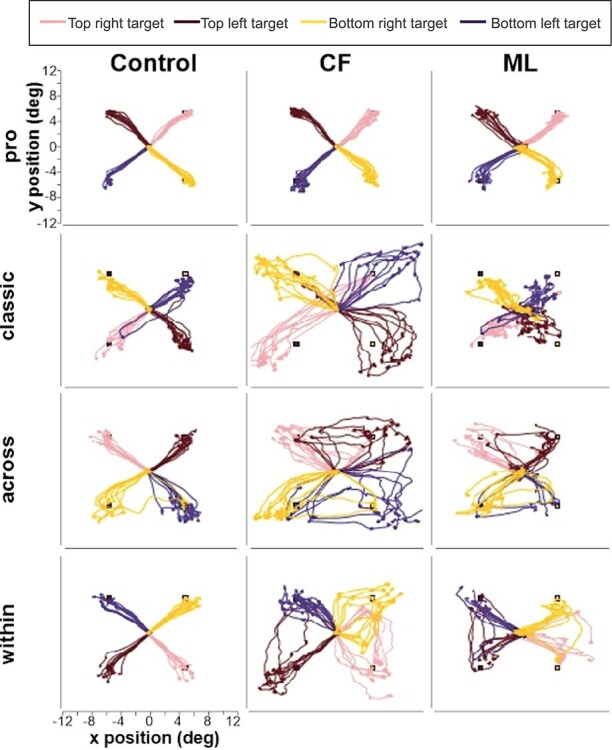
Raw saccade traces. Example saccade traces are shown separately for each patient and a typical control for each of the 4 paradigms. Saccade traces are color coded for each visual target location: pink for the top right target, brown for the top left target, yellow for the bottom right target, and dark blue for bottom left target.

In Figure 3 are plotted the raw eye movement traces for 10 randomly selected trials for each target for each patient as well as one typical control participant for all 4 conditions, randomly selected from the set of trials. Target locations and their corresponding saccades are color coded. There were several observations made from these traces that were investigated in more detail in subsequent analyses. First, in the pro-saccade condition, patients showed a slight increase in variability compared to the controls (in the left hemifield: blue and brown traces). In addition, for the classic and mirror saccades (i.e., across and within conditions), patients showed a large increase in variability and appeared to need more saccades than controls to reach the intended goal. They particularly seemed to make more variable or erroneous saccades when the visual target was presented in the left hemifield (blue and brown traces); these were corrected with subsequent saccades.

### Error Rates

We then compared mean ERs for patients and their age-matched controls. For all patients, we compared ERs collapsed across hemifields to their control groups. We also repeated the analyses separately by visual target hemifield. We then investigated whether the difference between their ERs for targets presented in their left and right hemifield differed from that observed for controls (these results are illustrated in Supplementary Fig. 1A and reported in Table 1).

**Table 1:** ERs per saccade condition

	Across				Within				Classic			
Participants	Mean ER (%)	SEM (%)	*df*	*t*	Mean ER (%)	SEM (%)	*df*	*t*	Mean ER (%)	SEM (%)	*df*	*t*
C.F.												
Left	42.31				25.00				17.74			
Right	30.77				37.29				17.39			
Controls												
Left	9.97	4.28	5	2.86^*^	10.49	3.65	5	1.50	9.09	2.93	4	1.21
Right	11.50	5.77	5	1.26	13.07	4.03	5	2.27	7.41	4.14	4	0.98
Left versus right			5	1.92			5	1.40			4	0.17
M.L.												
Left	31.82				25.81				33.33			
Right	9.09				17.24				14.89			
Controls												
Left	10.60	6.00	7	1.18	15.91	7.11	7	0.46	9.05	3.98	7	2.04
Right	7.85	4.88	7	0.09	12.44	4.73	7	0.34	6.55	2.94	7	0.95
Left versus right			7	4.20^**^			7	0.20			7	3.11^**^

*Note.* df, degree of freedom; SEM, standard error of the mean; *t* indicates results from modified *t*-tests (Crawford and Howell 1998; Crawford and Garthwaite 2002; Crawford et al. 2010).

^*^P < 0.05.

^**^P < 0.01.

In general, there was no significant difference between C.F. and controls’ ERs; we noted no significant difference for hemifields tested bilaterally (*P* > 0.0947). However, there was a significant difference for left targets during the across paradigm where C.F. had significantly higher ERs (*P* = 0.018) while his performance for right targets did not significantly differ from controls (*P* = 0.262).

When tested bilaterally, M.L.’s ER also did not significantly differ from controls’ (*P* > 0.18). When tested unilaterally however, M.L. showed an overall significant asymmetry between hemifields with higher ERs for left visual targets for the across and classic anti-saccade conditions, but not for the within hemifield condition. For the across condition, M.L. showed no significant difference compared to controls for ERs during separate analyses for left, *P* = 0.277, and right targets, *P* = 0.935. However, when we examined the difference between left and right target performance for M.L. and controls, we found a significant difference, *P* = 0.004. This is explained by higher ERs for left (contralesional) targets in our patient compared to right (ipsilesional) targets. In the classic condition, we similarly found no significant difference between groups for left, *P* = 0.081, and right targets analyzed individually, *P* = 0.376. However, we observed significantly higher ERs for left target compared to right target for M.L., *P* = 0.017, while controls maintained comparable means for both targets.

These results suggest a pattern where patients showed impaired performance for left visual target.

### Saccade Reaction Times

Analyses showed consistent increases in SRTs for patients compared to controls (see Supplementary Fig. 1B and Table 2). We first tested patients with SRTs collapsed across hemifields before examining them separately. In doing so, we observed a difference in SRTs between left and right targets compared to controls in across and classic conditions.

**Table 2:** SRTs per saccade condition

	Pro				Across				Within				Classic			
Participants	Mean SRTs (ms)	SEM (ms)	*df*	*t*	Mean SRTs (ms)	SEM (ms)	*df*	*t*	Mean SRTs (ms)	SEM (ms)	*df*	*t*	Mean SRTs (ms)	SEM (ms)	*df*	*t*
C.F.																
Left	182.58				400.57				366.19				416.82			
Right	151.18				312.76				356.22				295.89			
Controls																
Left	202.21	23.15	5	−0.32	305.14	18.02	5	2.00	323.32	25.84	5	0.63	340.67	20.22	4	1.54
Right	201.70	20.53	5	−0.93	297.64	19.19	5	0.30	320.96	25.63	5	0.52	343.40	23.21	4	−0.84
Left versus right			5	2.18			5	5.28^*^			5	0.21			4	2.82^**^
M.L.																
Left	338.59				645.47				613.70				500.81			
Right	322.87				529.47				619.04				463.33			
Controls																
Left	233.74	13.93	7	2.51	307.96	15.52	7	7.25^***^	311.22	14.21	7	7.10^***^	305.46	11.93	7	5.50^***^
Right	238.01	11.87	7	2.28	302.12	13.15	7	5.76^***^	314.17	13.96	7	7.28^***^	304.98	13.89	7	3.80^*^
Left versus right			7	0.74			7	2.63^**^			7	0.37			7	5.00^*^

*Note. df*, degree of freedom; SEM, standard error of the mean; *t* indicates results from modified *t*-tests (Crawford and Howell 1998; Crawford and Garthwaite 2002; Crawford et al. 2010)

^*^P < 0.01.

^**^P < 0.05.

^***^P < 0.001.

For all C.F.’s SRTs evaluated bilaterally, we found no significant difference from controls (*P* > 0.419). When tested unilaterally, C.F.’s pro-saccades SRTs did not differ from controls for both left (*P* = 0.762) and right targets (*P* = 0.395), but the difference between left and right target SRTs for the patient tended to differ from that of controls (*P* = 0.081). The classic and the across conditions showed similar results: No difference in mean SRTs for targets analyzed separately (left, *P* = 0.102; right, *P* = 0.778 in the across condition; left, *P* = 0.199; right, *P* = 0.450 in the classic condition), but a significant difference in SRTs between target sides between patient and controls (*P* = 0.003 in the across condition; *P* = 0.048 in the classic condition). This was the result of higher SRTs for left targets compared to right targets for our patient compared to controls who had comparable means for both target sides.

M.L. showed higher SRTs across all conditions for targets examined both bilaterally (*P* < 0.041) and unilaterally. Specifically, her mean SRTs were significantly delayed compared to controls for left, *P* = 0.041, and right targets, *P* = 0.049, in pro-conditions, and even more so in anti-saccades conditions (*P* < 0.001). The difference between left and right target SRTs did also significantly differ between our patient and her controls for the across and the classic conditions (*P* = 0.034 and *P* = 0.002, respectively) with higher SRTs for left targets.

### ER**s as a Function of SRTs**

**Figure 4:**
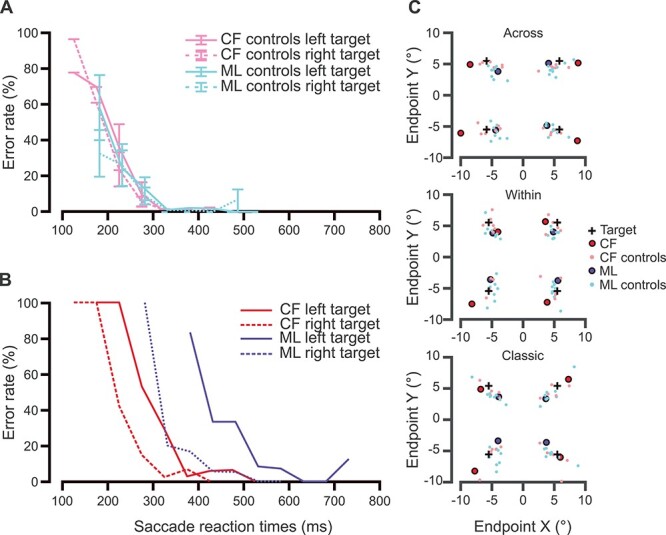
Temporal and spatial anti-saccade errors. In (*A*) and (*B*), we present anti-saccade ERs as a function of SRTs collapsed across anti-saccade conditions. Specifically, in (*A*), we show control groups’ ERs as a function of SRTs where C.F.’s controls are in pink and M.L.’s controls in pale blue. In (*B*), we illustrate patients’ data; C.F.’s in red and M.L.’s in blue. For both patients, solid lines were used for left targets while dotted lines were used for right targets. As seen in the first panel, controls had higher ERs for short SRTs (e.g., 100–200 ms window). These ERs then decreased sharply as SRTs became longer. Compared to controls, patients showed a delay in of the delay of ERs as a function of SRTs. In (*C*), we present mean saccade endpoints in *X* and *Y* degrees per target per anti-saccade condition. Possible target positions on the screen are marked with black crosses. In top panel, we show mean saccade endpoints for the across condition for each target. These means are presented as red circles for C.F. and blue ones for M.L. Controls’ endpoints are shown in pink and pale blue, respectively. Following the same color code, mean endpoints during the within paradigm are in the middle panel, and for the classic condition in the bottom panel.

Next, we investigated how ERs related to SRTs (Fig. 4*A*,*B*). Anti-saccade trials were collapsed across conditions. Overall, ERs declined as a function of SRTs, where highest ERs were found at short SRTs and lowest ERs at longer SRTs across all participants, with no difference between left and right targets for control subjects contrary to our patients. As described in Preliminary Analyses section, we conducted separated analyses for left and right targets, and we compared 20% ER rate thresholds between patients and controls (see Table 3). Ideally, we would have preferred to use a 0% ER, that is, the point at which no erroneous saccades were made, meaning that the visual target was perfectly inhibited. However, we selected a 20% threshold as opposed to 0% threshold due to the fact that patients did not seem to remain at 0% for longer SRTs (see Fig. 4*B*) and also to consider lapses, for example, participants made an erroneous saccade due to distraction.

**Table 3:** SRTs at 20% anti-ER threshold across patients and their control groups

Participants	SRTs (ms)	STDs (ms)	Diff left versus right(ms)	*df*	*t*
C.F.					
Left	324.74		69.71		
Right	255.03				
Controls					
Left	239.37	28.65	16.61	5	2.76^*^
Right	222.76	35.19		5	0.858
Left versus right				5	5.71^**^
M.L.					
Left	478.78		154.01		
Right	324.77				
Controls					
Left	217.95	53.31	-3.67	7	4,61^**^
Right	221.62	46.81		6	2,06
Left versus right				7	6.86^***^

*Note*. df, degree of freedom; Diff; difference; SRT, saccade reaction time when anti-saccade ERs reached 20%; STD, standard deviation across control SRTs; *t* indicates results from modified *t*-tests (Crawford and Howell 1998; Crawford and Garthwaite 2002; Crawford et al. 2010).

^*^P < 0.05.

^**^P < 0.01.

^***^P < 0.001.

Controls’ ERs as a function of SRTs are depicted in Figure 4*A*. Across all control groups, controls showed high ERs at short SRTs (i.e., before 200 ms). This was followed by a rapid decrease in ERs between 200 and 300 ms before reaching a 0% ER for longer SRTs. In summary, this tendency shows difficulties inhibiting automatic saccades to the visual target for extremely short latencies, inferior to mean SRTs.

In Figure 4*B*, we showed ERs as a function of SRTs for C.F. for each hemifield separately in red. Data from controls (in pink in Fig. 4*A*) show overlapping data for the 2 targets (left in solid lines and right in dotted lines) with a sharp decline in ERs as SRTs increased. C.F.’s ERs appear to decline in a similar manner as controls, where shorter SRTs have higher ERs, and longer SRTs, lower ERs. However, C.F.’s results for left targets appear to be shifted to the right, showing a slower decline in ERs as a function of SRTs. C.F. reached 20% ER after a significant delay of roughly 70 ms compared to controls for targets in his affected hemifield (i.e., left), *P* = 0.04, while the threshold for right targets did not differ from controls, *P* = 0.43. The difference in threshold SRT between target sides was significant, showing a greater delay for the left hemifield compared to the right hemifield in our patient, while controls only showed a difference of 17 ms between hemifields, *P* = 0.002.

We observed an overall shift to the right for M.L.’s performance (in dark blue in Fig. 4*B*) compared to controls (in pale blue in Fig. 4*A*) as a result of longer SRTs for the patient. As previously, controls showed higher ERs for shorter SRTs and lower ERs for higher SRTs. ERs also declined more rapidly for controls compared to M.L. Further, M.L. was the patient who showed the largest delay compared to her control group; she reached an anti-ER 20% threshold at 258 ms after controls did for left targets, *P* = 0.002. Her threshold for right targets occurred at the 324 ms mark and showed a tendency to be delayed compared to controls, *P* = 0.08. Difference analyses between SRTs for her left and right targets compared to controls, showed a significantly greater delay for left targets, *P* = 0.0002. In contrast, controls’ SRT thresholds differed by 4 ms between hemifields.

Taken together, patients took longer to inhibit saccades to the visual target compared to controls. Importantly, as illustrated in Figure 4*B*, both patients C.F. and M.L. showed significantly shifted data for left compared to right targets.

### Absolute Errors in Saccade Endpoints

To investigate bias relative to visual target, we considered both absolute errors and saccade endpoints. Overall, absolute errors for correct anti-saccades did not differ from controls for any saccade condition including pro-saccades for M.L., *P* > 0.05. C.F. showed more imprecise saccade than his controls in both hemifields in the across condition (left, *P* = 0.011; right, *P* = 0.003). We report the details of these analyses in Table 4. These results overall suggest that patients are accurate.

**Table 4:** Absolute errors per saccade condition

	Pro				Across				Within				Classic			
Participants	Mean (°)	SEM (°)	*df*	*t*	Mean (°)	SEM (°)	*df*	*t*	Mean (°)	SEM (°)	*df*	*t*	Mean (°)	SEM (°)	*df*	*t*
C.F.																
Left	1.29				4.41				3.31				3.29			
Right	1.10				4.42				3.04				3.87			
Controls																
Left	1.04	0.21	5	0.45	2.01	0.23	5	3.90^*^	2.26	0.39	5	1.01	2.67	0.35	4	0.73
Right	1.17	0.22	5	−0.12	1.99	0.17	5	5.49^**^	2.29	0.42	5	0.68	2.39	0.35	4	1.71
Left versus right			5	0.94			5	1.87			5	0.48			4	1.37
M.L.																
Left	0.99				1.99				2.05				3.07			
Right	1.21				2.09				1.81				2.79			
Controls																
Left	1.38	0.13	7	−0.99	2.40	0.16	7	−0.86	2.29	0.19	7	−0.42	2.82	0.34	7	0.25
Right	1.56	0.09	7	−1.32	2.63	0.27	7	−0.68	2.34	0.23	7	−0.77	2.58	0.20	7	0.35
Left versus right			7	0.32			7	0.25			7	0.30			7	0.19

*Note*. df, degree of freedom; SEM, standard error of the mean; mean and SEM are expressed in visual degree angles; *t* indicates results from modified *t*-tests (Crawford and Howell 1998; Crawford and Garthwaite 2002; Crawford et al. 2010).

^*^P < 0.05.

^**^P < 0.01.

Further, we found that saccade endpoints were within the distribution of controls. We determined where participants’ saccades landed relative to the saccade goal and visual target locations in the anti-conditions. In Figure 4*C*, mean saccade endpoints are shown for the patients and their controls. It can be noted that for all anti-conditions, the mean endpoints tended to be aligned to the visual target-saccadic goal vector for all participants but exaggerated in the patients. For the across condition, the visual target was opposite across the vertical meridian, and it can be seen that the endpoints tended to lie in this direction (stretched horizontally). In contrast, in the within condition, the visual target was opposite across the horizontal meridian, and the endpoints tended to be stretched vertically. Finally, in the classic condition, the visual target was diagonally opposite, and the endpoints were stretched diagonally. M.L.’s endpoints tended to be closer to the visual target while C.F.’s endpoints were biased away from the visual target past the saccade goal location.

### Saccade Correction

Finally, we examined whether patients and controls corrected their saccades after an erroneous anti-saccade (Table 5). This served to confirm that all participants understood task instructions. For patients as well as controls, erroneous anti-saccades (i.e., saccade to visual target during anti-saccade trials) tended to be further corrected toward the expected saccade goal. Taken together, this shows that all participants understood the task’s instructions.

**Table 5:** Proportion of corrected saccades following an erroneous anti-saccade

	Across			Within			Classic		
Participants	Number of incorrect	Mean (%)	SEM (%)	Number of incorrect	Mean (%)	SEM (%)	Number of incorrect	Mean (%)	SEM (%)
C.F.									
Left	22	72.73	0	14	85.71	0	11	63.64	0
Right	24	83.33	0	22	86.36	0	12	100	0
Controls									
Left	23	98.72	1.28	28	62.96	15.56	29	66.67	11.79
Right	30	88.61	9.63	29	74.05	15.35	23	70.24	16.87
M.L.									
Left	7	57.14	0	8	87.50	0	13	61.54	0
Right	3	100	0	5	100	0	7	57.14	0
Controls									
Left	30	61.90	18.87	45	64.86	12.95	43	81.75	7.70
Right	22	64.67	19.54	40	75.07	8.55	28	60.14	13.71

*Note.* The number of incorrect saccades refers to absolute number of erroneous anti-saccades. Means and SEM are expressed in percentages of corrected anti-saccades.

## Discussion

We tested how dorsal PPC damage affects anti-saccade production in 2 unilateral optic ataxia patients. Patients’ saccade endpoints did not differ from controls, showing that they were accurate, even though they appear to show an exaggerated bias along the visual target-saccade goal vector, M.L. appeared to undershoot targets while C.F. tended to overshoot them. Crucially, our patients showed delayed spatial inhibition of the visual target and made more erroneous saccades to it when it was contralateral to their lesions. Their performance was also particularly degraded for anti-saccade conditions where the visual target and intended saccade goal were in opposite hemifields (across and classic conditions). We interpret this pattern of results as demonstrating a specific role of the dorsal part of the posterior parietal cortex in spatial inhibition of the contralateral visual target for interhemispheric remapping.

We used a blocked paradigm to limit the influence of other factors such as switch costs associated with the interleaved pro/anti-saccade paradigms (Munoz and Everling 2004; Weiler and Heath 2012a; Weiler and Heath 2012b). Since blocked paradigms only require the cognitive demand of maintaining the same task instructions during the entire block, they may more directly measure inhibitory abilities compared to interleaved paradigms (Ethridge et al. 2009). Task switching has also been linked to proactive inhibition, which would have affected the interpretation of our results since we were mainly interested by spatial inhibition.

We observed impairments in anti-saccade production for our patients specifically when the visual target was presented in the contralesional visual field. This is consistent with spatial inhibition deficits rather than response or proactive inhibition. If response inhibition processes had been impaired, our patients would have shown increased anti-saccade errors independent of the hemifield in which the visual target was presented, such as has been reported in neglect patients with unilateral lesions (Butler et al. 2009) and patients with frontal lesions (Guitton et al. 1985). Similarly, if proactive inhibition had been impaired, we would not have observed asymmetric effects as we did.

Deficits in visual working memory also cannot fully explain the present results. Working memory has been proposed to be closely related to attention (Kane et al. 2001; Chuderski 2014; Shipstead et al. 2015; Meier et al. 2018), with both constructs linked to performance in anti-saccades (Walker et al. 1998; Unsworth et al. 2004; Magnusdottir et al. 2019). However, working memory has been more closely associated with the general ability to suppress an automatic response to the target, that is, influencing ERs “bilaterally,” and less so with the ability to make anti-saccades, that is, influencing SRTs (Norman and Shallice 1986; Roberts et al. 1994; Bjorklund and Harnishfeger 1995; Mitchell et al. 2002; Eenshuistra et al. 2004; Magnusdottir et al. 2019). Our results point to specific slowing and delayed SRTs for correct anti-saccades in our patients instead of increased anti-saccades ERs. Accordingly, working memory has been more specifically associated to hemineglect following right-hemispheric ventral network (Pisella et al. 2004; Pisella et al. 2015; Pisella 2017), and optic ataxia patients do not present working memory deficits (Valdois et al. 2019).

Several points support the notion that spatial inhibition processes are affected in our patients; we observed both higher ERs and more delayed SRTs when the visual target was presented in ataxic hemifields. This suggests that patients were impaired in the inhibition of the visual target when it was present in their affected hemifields. During our temporal analysis collapsed across conditions, we highlighted this impairment further where both M.L. and C.F. took longer to reach 20% ER for visual target presentation in their affected hemifield compared to their unaffected hemifield and to controls.

The delayed spatial inhibition processes observed in our patients could be explained by perturbed priority maps following dorsal PPC damage. The PPC has been implicated in attentional processes, particularly with respect to priority maps (Fecteau and Munoz 2006; Serences and Yantis 2006; Bisley and Goldberg 2010; Mirpour et al. 2010). Priority maps are map-like representations in the brain where an object’s salience and the observer’s goals or motivation (i.e., relevance) interact to determine attentional allocation as well as saccade goal selection (Gold and Shadlen 2000; Itti and Koch 2001; Fecteau and Munoz 2006; Serences and Yantis 2007; Franconeri et al. 2013). On these maps, representations of visual stimuli compete in a winner-takes-all rule; the representation of an object or location with the highest level of neuronal activity receives the highest priority for saccade planning, whereas irrelevant object-related activity is suppressed (Koch and Ullman 1985). During target selection, neuronal activity changes dynamically until activation for a given object is proportional to its behavioral priority (Schall et al. 1995; Gold and Shadlen 2000; Itti and Koch 2000; Fecteau and Munoz 2006; Goldberg et al. 2006; Serences and Yantis 2007; Armstrong et al. 2009; Bisley and Goldberg 2010). The facilitation of a target selection by its enhanced activity is simultaneously accompanied by the suppression of the response associated with irrelevant competing objects, which gives the advantage to the prioritized target (Egeth and Yantis 1997; Kastner et al. 1998; Reynolds et al. 1999; Serences et al. 2004). During anti-saccades, it would be expected that the spatial representation of the saccade goal receives the highest priority as it is relevant to the task, while the visual target, which is salient but irrelevant, is suppressed. Previous findings have showed that the time required to inhibit a salient object (i.e., visual target here) affects saccade planning (Wolf and Lappe 2020); inhibition is a dynamic process that requires time to dampen saliency-related activity and enhance goal-related activity (Schall et al. 1995; Gold and Shadlen 2000; Itti and Koch 2000; Fecteau and Munoz 2006; Goldberg et al. 2006; Serences and Yantis 2007; Armstrong et al. 2009). Delays in spatial inhibition of the visual target due to PPC damage would thus result in higher ERs and anti-SRTs in affected hemifields as seen in our patients.

Patients’ performance was also particularly degraded for anti-saccade conditions where the visual target and intended saccade goal were in opposite hemifields (across and classic conditions), and less so for the within condition where target and saccade goal were in the same hemifield. An imaging study using between-hemifields anti-saccades showed that the PPC encodes both the saccade goal and target location (Medendorp et al. 2005)—initial activity representing the visual target in one hemisphere was transferred through a dynamic shift across hemispheres for the saccade goal representation. It is possible that unilateral damage to the symmetrical dorsal PPC network impedes this interhemispheric shift of neuronal activity required when the visual target and saccade goal are in separate hemifields as it is the case for our across and classic anti-saccade conditions. However, this cannot explain the asymmetrical results found in our patients; they had shorter SRTs and smaller ERs when the target was in the ipsilesional hemifield (i.e., right target) and the saccade goal in the contralesional hemifield (i.e., left saccade), than the opposite (left target and rightward saccade). For this reason, the vector inversion cannot solely involve a dynamic shift of activity at the level of the symmetrical network of the dorsal PPC for anti-saccades.

This asymmetry may be related to previous PPC lesion work suggesting a dominance of the right hemisphere for mental rotation (Harris and Miniussi 2003) and remapping processes (Pisella et al. 2011). The right IPL would contain a bilateral priority map where all visual targets and saccade goals can be represented. This right-hemispheric map transfers the result of the remapping to oculomotor structures (i.e., frontal eye field, supplementary eye field, dorsolateral prefrontal cortex, pre-supplementary motor area; Connolly et al. 2002; Curtis and D'Esposito 2003; Ford et al. 2005; Fernandez-Ruiz et al. 2007) where saccade goal is transformed into a motor plan (Pisella et al. 2011). One could thus postulate that in optic ataxia patients, damage to the right dorsal PPC would prevent the transfer of competing information (i.e., left visual target and right saccade goal representations) toward the right IPL for left-to-right remapping. In contrast, the (spared) left dorsal PPC would transfer the competing information (right visual target and left saccade goal representations) toward the spared right IPL for right-to-left remapping. Further, the right IPL would not require any transfer from the lesioned right SPL for intrahemispheric remapping of left targets, such as during the within paradigm.

In summary, our results show that unilateral dorsal PPC lesion affects the inhibition of the contralesional visual target representation especially when interhemispheric remapping is required. Spatial inhibition and remapping can be linked in priority maps, where attention and remapping processes are additive processes that may rely on bilateral dorsal PPC network and right IPL, respectively (Melcher 2009; Pisella et al. 2015). During anti-saccades, the intentional remapping/mental rotation processes would establish a new neuronal representation at the appropriate saccade goal location on a right-hemispheric priority map. For interhemispheric remapping, the 2 representations of the visual target and saccade goal would compete for attention allocation (Egeth and Yantis 1997; Kastner et al. 1998; Reynolds et al. 1999; Serences et al. 2004) on the symmetrical dorsal PPC priority maps and the hemispheric side where the visual target is inhibited would transfer the competition resolution to the right IPL for further anti-saccade planning.

Overall, we showed deficits in anti-saccade production in optic ataxia patients. Taken together, our results point to a specific role of the dorsal PPC in the spatial inhibition processes underlying anti-saccades across hemifields, resulting in impaired and delayed resolution of competing saccade vectors.

## Supplementary Material

S1_legend_tgab054

SupplementaryFig1_tgab054

## Notes

We would like to thank our 3 patients and control participants for their time and contribution, as well as Michelle Soulier, Sonia Alouche, Jean-Louis Borach, and Anton Malienko for their administrative and experimental assistance. *Conflict of Interest*: All authors declare that they have no conflict of interest.
