# Supplementary material for: Impaired Spatial Inhibition Processes for Interhemispheric Anti-saccades following Dorsal Posterior Parietal Lesions
Source: Cereb Cortex Commun. 2021 Sep 13;2(3):tgab054. doi: 10.1093/texcom/tgab054 (PMC8481671; doi:10.1093/texcom/tgab054)
Supplement: S1_legend_tgab054 [file s1_legend_tgab054.docx]

S1. Overall anti-saccade performance for patients and their age-matched controls. Panel A shows mean error rates for patients and their age-matched controls across conditions tasks. C.F.’s error rates (ER) are shown in in red, and M.L.’s in blue. Dark red or dark blue represent ERs for targets presented in the left hemifield while pale red or pale blue illustrate right targets. In grey, we depict 95% confidence intervals for controls with their mean as a black line. We compared each patient with their respective control group using modified t-tests, * = p < .05, ** = p < .01. In B, we present mean saccade reaction times across patients across conditions tasks. C.F.’s saccade reaction times (SRT) are in red, and M.L.’s in blue. Dark coloured bars represent SRTs for targets presented in the left hemifield while pale coloured ones represent right targets. In grey, we depict 95% confidence intervals for each patient’s controls with their mean as a black line. We compared patient with their respective control group with modified t-tests, * = p < .05, ** = p < .01, *** = p < .001
